# The combination of sorafenib and everolimus shows antitumor activity in preclinical models of malignant pleural mesothelioma

**DOI:** 10.1186/s12885-015-1363-1

**Published:** 2015-05-08

**Authors:** Ymera Pignochino, Carmine Dell’Aglio, Simona Inghilleri, Michele Zorzetto, Marco Basiricò, Federica Capozzi, Marta Canta, Davide Piloni, Francesca Cemmi, Dario Sangiolo, Loretta Gammaitoni, Marco Soster, Serena Marchiò, Ernesto Pozzi, Patrizia Morbini, Maurizio Luisetti, Massimo Aglietta, Giovanni Grignani, Giulia M Stella

**Affiliations:** 1 Division of Medical Oncology, IRCCS-Institute for Cancer Research and Treatment, Candiolo, (TO), 10060 Italy; 2 Department of Molecular Medicine, − Section of Pneumology, Laboratory of Biochemistry & Genetics, University and Fondazione IRCCS Policlinico San Matteo, Pavia, 27100 Italy; 3 Laboratory of Tumor Microenvironment, IRCCS-Institute for Cancer Research and Treatment, Candiolo, (TO), 10060 Italy; 4 Department of Molecular Medicine- Section of Pathology, University of Pavia and Fondazione IRCCS Policlinico San Matteo, Pavia, 27100 Italy

**Keywords:** mTOR, ezrin, Malignant pleural mesothelioma, Targeted therapy, Preclinical models, Apoptosis, Reactive oxygen species, Translational oncology

## Abstract

**Background:**

Malignant Pleural Mesothelioma (MPM) is an aggressive tumor arising from mesothelial cells lining the pleural cavities characterized by resistance to standard therapies. Most of the molecular steps responsible for pleural transformation remain unclear; however, several growth factor signaling cascades are known to be altered during MPM onset and progression. Transducers of these pathways, such as PIK3CA-mTOR-AKT, MAPK, and ezrin/radixin/moesin (ERM) could therefore be exploited as possible targets for pharmacological intervention. This study aimed to identify ‘*druggable*’ pathways in MPM and to formulate a targeted approach based on the use of commercially available molecules, such as the multikinase inhibitor sorafenib and the mTOR inhibitor everolimus.

**Methods:**

We planned a triple approach based on: i) analysis of immunophenotypes and mutational profiles in a cohort of thoracoscopic MPM samples, ii) *in vitro* pharmacological assays, ii) *in vivo* therapeutic approaches on MPM xenografts. No mutations were found in ‘hot spot’ regions of the mTOR upstream genes (e.g. EGFR, KRAS and PIK3CA).

**Results:**

Phosphorylated mTOR and ERM were specifically overexpressed in the analyzed MPM samples. Sorafenib and everolimus combination was effective in mTOR and ERM blockade; exerted synergistic effects on the inhibition of MPM cell proliferation; triggered ROS production and consequent AMPK-p38 mediated-apoptosis. The antitumor activity was displayed when orally administered to MPM-bearing NOD/SCID mice.

**Conclusions:**

ERM and mTOR pathways are activated in MPM and ‘*druggable’* by a combination of sorafenib and everolimus. Combination therapy is a promising therapeutic strategy against MPM.

**Electronic supplementary material:**

The online version of this article (doi:10.1186/s12885-015-1363-1) contains supplementary material, which is available to authorized users.

## Background

Malignant Pleural Mesothelioma (MPM) is an aggressive tumor characterized by poor prognosis and by continuously increasing incidence due to widespread exposure to asbestos [1]. There is still no effective therapeutic regimen for MPM; as a consequence, the median survival is approximately one year [2]. This tumor therefore represents an unsolved health problem with an urgent medical need.

MPM is histologically classified as (i) epithelial (50-70% of cases), (ii) mesenchymal or sarcomatous (7-20% of cases), and (iii) mixed or biphasic (20-35% of cases) [3]. Although most of the molecular steps driving MPM onset and progression are still unclear, several signaling pathways are known to be altered in this disease [4]. Among them, activated phosphatidyl inositol-3-kinase-mammalian target of rapamycin-protein kinase B (PIK3CA-mTOR-AKT) [5,6] and mitogen-activated protein kinase (MAPK) [7] cascades have been documented as playing a relevant role in MPM progression, being further associated to a worst prognosis [8]. However, small molecule inhibitors of the upstream tyrosine kinase receptors (e.g. the epithelial growth factor receptor, EGFR), although successfully used for the treatment of different epithelial tumors, are not effective against MPM [9,10]. A better understanding of MPM biology is therefore a clear priority both for the assessment of targeted agents, and for the selection of patients that are likely to achieve clinical benefit. We here report a deep investigation of the main oncogenic pathways that could be involved in MPM with particular interest in mTOR pathway. Moreover, unlike many other cancers, MPM progression does not generally impact distant organs, but normally affects the organs around the pleura, mainly the lungs on the side of the body in which the original tumor was found and the abdomen and peritoneal cavity. From this basis, we hypothesized that activation of ezrin-radixin-moesin (ERM) members of the cell cortex involved in cell adhesion, cell motility, and signal transduction [11,12] could have a role in inducing the peculiar pattern of ‘local spread’ in MPM. Overall this approach was followed by the design and testing of therapeutic strategies targeting the mTOR and the ERM signaling cascade. mTOR is a kinase of the PIK3CA-related family acting as key regulator of the switch between catabolic and anabolic metabolism. In the last decade, this transducer has emerged as a therapeutic target for a number of diseases, among which cancer [13]. We therefore reasoned that molecular lesions affecting the mTOR signaling pathway could be *targettable* by clinical grade, mTOR-specific inhibitors. The plasma membrane-cytoskeleton linker protein ezrin, member of ERM family, plays a crucial role in the proliferation and metastatization of several aggressive tumors, being sarcomas the most investigated [14,15]. Ezrin is activated by phosphorylation during cell growth and motility in both normal and tumor-derived cells [16]. We have previously demonstrated for the first time that this pathway can be therapeutically targeted in preclinical models of osteosarcomas, by treatment with the multi-kinase inhibitor sorafenib. In particular this drug to inhibits the phosphorylation of ERM in their critical sites (Thr567 on ezrin; Thr564 on radixin and Thr558 on moesin) [17]. However, so far the role of ERM proteins in MPM has never been investigated.

To identify novel, molecularly-targeted therapeutic strategies immediately available for clinical use in MPM management, we here explored the *in vitro* and *in vivo* anti-tumor effects of the mTOR specific inhibitor everolimus in combination with sorafenib. Although both drugs are already in phase II clinical trial for MPM, no data are available concerning the effects of the association.

In this work, for the first time we show that ERM proteins are activated in MPM and ezrin in particular has a role in cell proliferation and migration of MPM cells. Moreover, we demonstrated that sorafenib and everolimus combination has antitumor activity in MPM preclinical models *in vitro* and *in vivo.*

## Methods

### MPM sample selection

We selected and analyzed 30 MPM samples (10 cases for each histological subtype) derived from medical thoracoscopy from a cohort of patients aged ≥18 years who referred to the Pneumology Department at Fondazione IRCCS Policlinico San Matteo, Pavia, Italy. The histopathological analysis of sections from formalin-fixed, paraffin-embedded (FFPE) specimens was performed at the Pathology Unit of the same hospital. Complete clinical data of each patient object of this study are listed in Additional file 1: Table S1.

The expression of phospho-m-TOR (P-mTOR) and phospho-ERM (P-ERM) proteins was investigated by immunohistochemistry, as described in the Additional file 1.

Tumor DNA was extracted from each FFPE sample with a commercial kit following the manufacturer’s recommendations; in all cases the histological sections contained more than 80% tumour tissues and less than 5% necrosis. We evaluated the mutational profile of ‘hot spot’ regions of three oncogenes frequently mutated in solid cancers: KRAS (exon 2), EGFR (exons 18-19-20-21) and PIK3CA (exons 9–20), as described in the Additional file 1.

### MPM cell culture, drugs and reagents

The human MPM cell lines MSTO-211H, NCI-H28, NCI-H2052 and NCI-H226 were obtained from the American Type Culture Collection (Rockville, MD). Ist-Mes-1 and Ist-Mes-2 were obtained from Genoa Institute Culture Collection. Two cell lines (MM001, MM002) were established in our laboratory from pleural effusions derived from two patients diagnosed with MPM and characterized by routine pathology evaluation. The procedure was approved by the local Ethical Commission and each enrolled patient gave written informed consent before enrolment (Comitato di Bioetica, Fondazione IRCCS Policlinico San Matteo, approval numbers: protocol #20090002344; procedure #20090019080; date of approval: June 3rd, 2009). MES-MM98 cell line was from the biobank of the Hospital of Alessandria (Pathology Unit), and previously described [18]. Cells culture protocols are described in the Additional file 1. Sorafenib (SOR) and everolimus (EV) (Sequoia Research Product, UK) stock solutions were prepared in DMSO and stored at −20°C. Control and human ezrin-specific siRNA were purchased from Ambion (Life Technologies Italia, Monza MB).

### Biological and biochemical assays

Each MPM cell line was incubated with scalar doses of sorafenib (from 10 μM to 0.625 μM), everolimus (from 2 μM to 12.5 nM), or their constant combination. Proliferation, migration, and colony formation capacity were evaluated to assess differences between (i) treated and untreated cells, and (ii) control and human ezrin-specific siRNA-transduced cells. Details about the *in vitro* assays are listed in the Additional file 1.

To analyze the activation of signal transduction cascades, MPM cells (80% confluence) were treated for 24 h with EV (100 nM) or SOR (5 μM), alone or in combination, or left untreated. Western Blot analysis is described in the Additional file 1.

The impact of cell treatment on apoptosis, cell cycle, radical oxygen species (ROS) production was analyzed by assays based on flow cytometry as described in the Additional file 1.

### Mice xenograft models

Non-obese diabetic/severe combined immunodeficient (NOD/SCID) female mice (Charles River, Milan, Italy) were breed, maintained in cage microinsulators, and handled under sterile conditions at the animal facility of the Institute for Cancer Research and Treatment (Candiolo, Italy). Animal manipulation was approved by the Institute’s Ethical Commission and by the Italian Ministry of Health. In three independent experiments, 24 female mice (4–6 weeks old) were injected subcutaneously (s.c.) into the right flank with 10^6^ MSTO-211H cells in growth factor-reduced BD Matrigel basement membrane matrix (BD Biosciences, San Jose, CA). When xenografts were established at about 100 mm^3^ after 5 weeks, animals were divided into 4 groups and were treated daily by oral gavage with either sorafenib (5 mg/kg/die), everolimus (1 mg/kg/die), their combination (sorafenib 5 mg/kg/die + everolimus 1 mg/kg/die), or vehicle alone for 4 weeks before the sacrifice (see Additional file 1).

### Ethics statement

The procedure was approved by local Ethical Commission and each enrolled patient gave written informed consent before enrolment (Comitato di Bioetica, Fondazione IRCCS Policlinico San Matteo, approval numbers: protocol #20090002344; procedure # 20090019080; date of approval: June 3rd, 2009). Mice experiments were approved by the Ethical Commission of the Institute for Cancer Research and Treatment (Candiolo, Torino, Italy), and of the Italian Ministry of Health.

## Results

### mTOR and ERM are activated in human MPM

**Figure 1:**
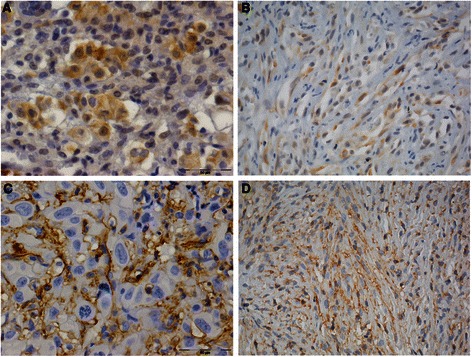
Immunohistochemical evaluation of the presence and amounts of P-ERM and P-mTOR in MPM samples. Representative fields of epitheloid **(A, C)** and sarcomatous **(B, D)** tumor subtypes expressing P-mTOR **(A, B)** and P-ERM **(C, D)** are shown. P-mTOR is present in the cytoplasm of malignant mesothelia clusters. P-ERM are diffusely and uniformly expressed on the membrane of neoplastic mesothelial cells.

Of the 30 mesothelioma specimens analyzed, 10 exhibited epithelioid, 10 biphasic and 10 sarcomatous histology. This cohort analyzed in the work derived by selection of 10 MPM cases of each major histological subtype and, thus, does not reflect the biology of MPM. All the patients referred occupational or environmental exposure to asbestos; the vast majority (25 out of 30) of the evaluated patients were previous and current smokers. Five out of the 30 patients were females. The median age at diagnosis was 67 years and the median overall survival (OS) was 15.9 months, coherent with already published data. We evaluated the expression of phosphorylated mTOR (P-mTOR) and ERM (P-ERM) by immunohistochemistry. P-mTOR was expressed in most cases (83%) in both sarcomatous and epithelial subtypes. P-ERM was present at moderate-high levels in almost all samples (93%), and was not associated to a particular histotype (Additional file 1: Table S1). Staining obtained in representative cases of epithelioid and sarcomatous MPM is shown in Figure 1. These results were confirmed by semi-quantitative evaluation (Additional file 1: Table S1). Within the limit of the cohort analyzed, the correlation between OS and intensity of P-mTOR seems to be not significant; whereas P-ERM really positive (+++) patients display a higher OS (Additional file 2: Figure S1). The analysis of mutational profiles in the ‘hot spot’ regions of EGFR (exons 18–21), KRAS (exon 2), and PIK3CA (exons 9 and 20) genes revealed no somatic mutations in MPM samples (Additional file 1: Table S1).

### Sorafenib and everolimus inhibit mTOR, ERK1/2 MAPK in MPM cell lines

**Figure 2:**
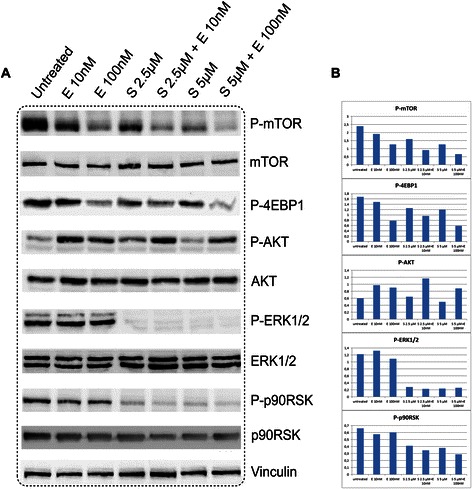
Inhibition of mTOR and ERK1/2 MAPK pathways by sorafenib (S), everolimus (E) and their combination. **A**. representative western blot images obtained with in MES-MM98 cells **B**. Histograms showing quantification of phosphorylated proteins after normalization with the respective total proteins and the housekeeping (vinculin).

The activation of the mTOR and ERK1/2 MAPK signalling cascades plays a fundamental role in cancer progression. Having shown that MPM samples expressed high amounts of phosphorylated mTOR (Figure 1), we investigated the druggability of these pathways by everolimus and/or sorafenib in preclinical models of MPM. As a first step in the design of a molecularly-targeted therapy, we treated seven different MPM cell lines with sorafenib and everolimus for 24 hours, followed by western blot evaluation of phosphorylated protein transducers. We confirmed a decreased phosphorylation of mTOR/4EBP1/p70S6K and MAPK/p90RSK signalling cascades in all cell lines treated with everolimus or sorafenib, respectively. As reported for other tumor types [19], we observed that everolimus induces AKT activation: this effect may be a result from a negative feedback loops involving p70S6K. Figure 2 show the results obtain in MES-MM98 as representative of 7 tested cell lines.

### Sorafenib and everolimus inhibit ERM phosphorylation and migration of MPM cell lines

**Figure 3:**
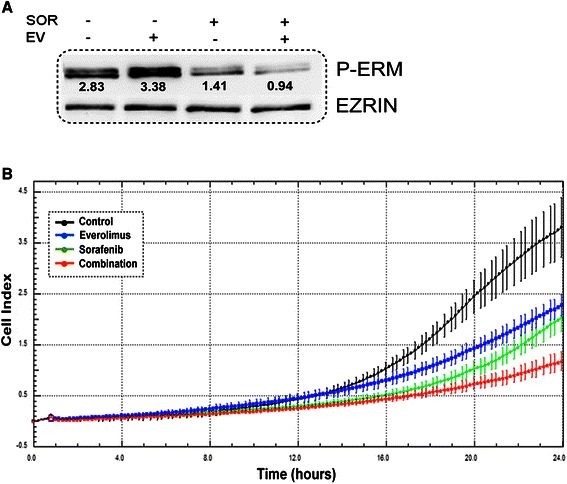
Sorafenib and everolimus effect on **(A)** ERM activation and **(B)** MES-MM98 cell migration; black line untreated cells, blue line everolimus 10nM, green line sorefenib 2.5 μM, and red line combination-treated cells.

**Figure 4:**
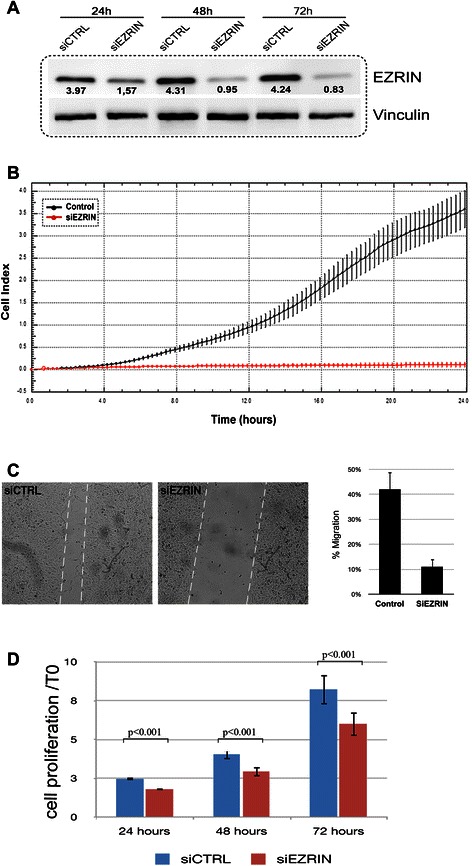
Silencing of ezrin in MES-MM98 cells. **A** western blot analysis of ezrin protein expression. **B**. *In vivo* time course XCelligence analysis of migrating H226 cells after 24 h from mock (control) and ezrin-specific siRNA treatment **C**. Wound healing assay with MPM cells after 72 hours from treatment with control siRNA (siCTRL), and ezrin-specific siRNA (siEZRIN). The histogram depicts cell migration, calculated as percentage of wound healing after 24 h from the scratch In the text: “We demonstrated that the down-regulation of ezrin slightly reduced cell viability after 24, 48, 72 hours (p < 0.001) and impinged on the migration ability of MPM cells **(Figure 4 B,C,D)**.

We have also shown that ERM proteins are broadly phosphorylated in MPM samples (Figure 1). Consequently, we evaluated the effect of everolimus and/or sorafenib on ERM activation in cultured MPM cells. We observed a slight increase in ERM phosphorylation after 24 h treatment with everolimus, whereas sorafenib caused a sharp decrease in ERM activation, both as a single agent and in combination (Figure 3A). Since ERM proteins and in particular ezrin are involved in signalling and cell migration in normal and cancerous cells [16] we evaluated the effect of sorafenib and everolimus in scratch assay and CELLingence migration test. We demonstrated that sorafenib and everolimus alone and even more in combination inhibited cell migration (p < 0.001, Figure 3B). To further elucidate the role of ERM proteins in MPM cells, we down-regulated the expression of its principal component, ezrin, by transducing H226 and MSTO-H211 with specific siRNA. After 48 and 72 h from silencing we observed a sharp reduction in the expression of this protein, as evaluated by western blot (Figure 4A). We demonstrated that the down-regulation of ezrin slightly reduced cell viability after 24, 48, 72 hours (p < 0.001) and impinged on the migration ability of MPM cells (Figure 4).

### Sorafenib and everolimus exert synergistic effects on MPM cell growth and viability

**Figure 5:**
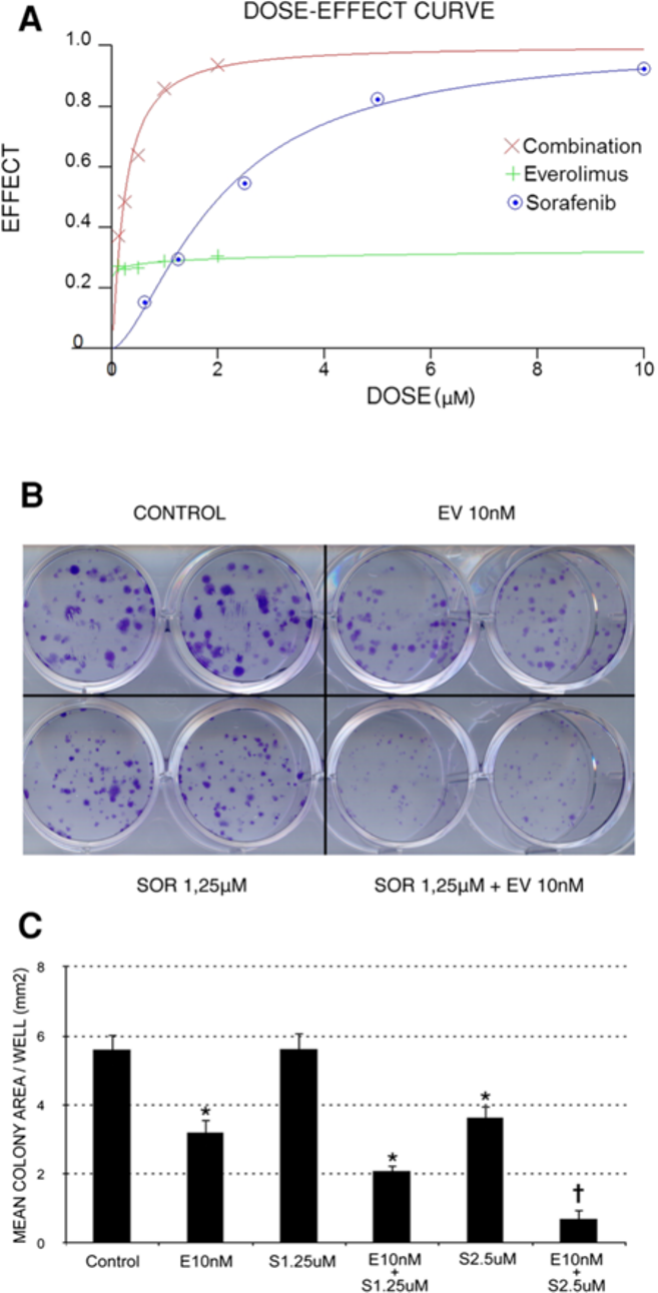
Synergistic anti-proliferative effects of sorafenib and everolimus on MES-MM98 cell lines, calculated according to the number of viable cells in culture based on quantitation of the ATP present. **(A)** Representative dose-effect curve of MES-MM98 cells treated with scalar doses of sorafenib, everolimus or their combination. The dose effect curve was calculated by CalcuSyn software after 72 h of treatment. **(B)** Photograph of a representative colony growth of MES-MM98 cells after 10 day-incubation in complete medium alone (NT) or with sorafenib (SOR 2.5 μM), everolimus (EV 10 nM), or their combination (SOR 2.5 μM + EV 10 nM). **(C)** Analysis of the total surface area occupied by colonies/well after treatment with escalating doses of sorafenib (0, corresponding to control, 1.25 μM and 2.5 μM), alone or in combination with everolimus (10 nM). *p < 0.05 *vs* NT; †, p < 0.05 *vs* both single agents and control. Results are expressed as mean ± standard deviation of triplicate experiments.

We then moved to investigate the biological readout of the inhibition of specific signalling pathways by everolimus and sorafenib in MPM cell lines. We evaluated whether sorafenib and everolimus influenced cell viability, by exposing cultured MPM cell lines to increasing doses of these two drugs, alone or in combination, for 72 hours. A measurement of cellular ATP content by means of cellTiterGlo® assay demonstrated that sorafenib induced a dose-dependent decrease in cell numbers in all tested MPM lines. Treatment with everolimus alone resulted in a weak effect, leading to a decrease in cell vitality from 20% to 30% at the highest tested dose (2 μM, Figure 5A). The treatment with the combination resulted in a significantly greater viability impairment compared to either agent alone, displaying synergism in the interval of 30-70% of fractions affected (CI < 1), as confirmed by a reduction of the IC50 for both drugs (Table 1). We next evaluated the capability of low doses of sorafenib, everolimus, and their combination, to interfere with cell growth *in vitro* after mid-long term culture. Figure 5C shows a representative experiment performed with MES-MM98 cells. In these assays, a 10 day-treatment with low-dose everolimus (10 nM) was sufficient to significantly reduce the mean colony area to 43 ± 6.8% of the control (p < 0.05). On the contrary, sorafenib alone displayed a significant effect only at 2.5 μM (35.2 ± 6.2% of the control. p < 0.05). A combined treatment resulted in a potentiated dose–response effect (62.8 ± 3.5% of the control with SOR 1.25 μM + EV 10 nM; 87.4 ± 4.9% of the control with SOR 2.5 μM + EV 10 nM, p < 0.05). The result obtained with the other MPM cell lines are shown in Additional file 3: Figure S2.

**Table 1:** **Drug concentration leading to a 50% inhibition of cell viability at 72 h (IC50) in cellTiterGLO® assay and Combination Index (CI) for each cell line**

MPM Cell Lines	IC50 Drug alone (95% confidence interval)		IC50 Drug combination (95% confidence interval)		CI at IC50 ± Est. st.dev*
	Everolimus (nM)	Sorafenib (μM)	Everolimus (nM)	Sorafenib (μM)	
MES-MM98	>100	4.21 *(2.91-6.09)*	40 *(10.6-150.5)*	2 *(1.03-4.02)*	*0.485 ± 0.20*
MM001	>100	2.01 *(1.86-2.21*)	23.6 *(14.9-37.5)*	1.18 *(0.75-1.87)*	*0.585 ± 0.08*
NCI-H226	>100	2.12 *(1.55-2.89)*	32 *(20.5-49.9)*	1.60 *(1.02-2.49)*	*0.755 ± 0.23*
MSTO-211H	>100	0.90 *(0.68-1.19)*	34 *(33.4-35.3)*	0.17 *(0.01-1.77)*	*0.426 ± 0.19*
IST-MES1	>100	1.69 *(1.10-2.60)*	28.3 *(17.1-46.8)*	1.42 *(0.85-2.34)*	*0.873 ± 0.30*
IST-MES2	>100	2.16 *(1.66-2.80)*	19.1 *(9.8-37.1)*	0.96 *(0.49-1.86)*	*0.573 ± 0.22*
NCI-H28	>100	3.34 *(2.27-4.92)*	27 *(14–51.6)*	1.36 *(0.71-2.58)*	*0.920 ± 0.34*
NCI-H2052	>100	6.21 *(4.45-8.66)*	44.7 *(31–63.9)*	2.23 *(1.56-3.19)*	*0.950 ± 0.21*
MM002	>100	2.78 *(2.30-3.35)*	18.6 *(6–57.2)*	0.93 *(0.30-2.86)*	*0.345 ± 0.12*

*combination index calculated at IC50 ± estimated standard deviation based on Chou-Talalay method.

### Everolimus potentiates the pro-apoptotic effect of sorafenib in MPM cell lines

**Figure 6:**
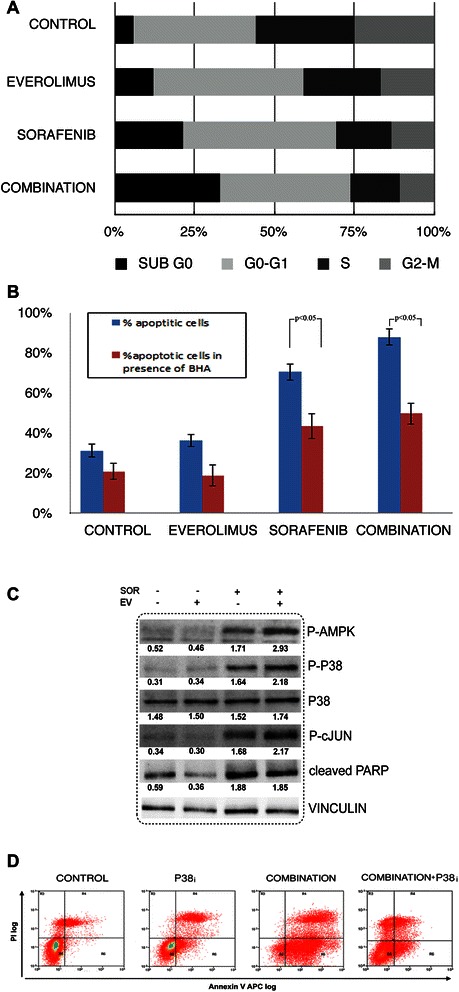
Mechanisms of apoptosis induction by sorafenib and everolimus combination. **(A)** Bar graph of cell cycle distribution in MES-MM98 cells after 48 h treatment with sorafenib (2.5 μM), everolimus (10 nM) and their combination, compared to untreated control. **(B)** Induction of apoptosis in MES-MM98 cells, in the absence (blue bars) or in the presence (red bars) of the ROS scavenger BHA. **(C)** Activation of AMPK by sorafenib treatment. Everolimus potentiated the effect of sorafenib on AMPK activation, reflecting the enhancement of mTOR inhibition and the pro-apoptotic effect of sorafenib and everolimus mediated by c-Jun and p38 activation, triggered by ROS production. PARP cleavage confirmed the apoptotic status. **(D)** Propidium iodide and Annexin V staining of MES-MM98 cells treated with sorafenib (2.5 μM), everolimus 10 nM, and the p38 inhibitor SB202190. These experiments are representative of 4 different tested cell lines

To investigate the mechanism(s) by which sorafenib and everolimus influenced the viability and/or proliferation of cultured MPM cells, we examined the cell cycle status by propidium iodide (PI) staining and cytofluorimetric evaluation after 48 hours of combined drug treatment (SOR 2.5 μM + EV 10 nM). We observed a decreased proportion of cells in the S and G2/M phase and an increased proportion of cells in the G0/G1 fractions, as a result of cell cycle arrest (Figure 6A). These analyses also revealed an increased percentage of drug-treated cells with sub-diploid DNA contents (32.61%) compared to single treatments (21.03% sorafenib, 11.81% everolimus) and control, vehicle-treated cells (5.6%). These data suggested that sorafenib and everolimus induced apoptosis in MPM cells. We therefore evaluated the percentage of apoptotic MPM cells by Annexin-V staining coupled to PI incorporation after 96 h from drug administration. As shown in Figure 6B, treatment of MES-MM98 cells with sorafenib alone (2.5 μM) significantly induced apoptosis (67.31% Annexin V/PI positive cells *vs* 25.99% in vehicle treated control cells). Everolimus alone had no effect on the percentage of apoptotic cells (27.69% Annexin V/PI positive cells). However, the combined drug treatment resulted in potentiated effect (85.42% Annexin V/PI positive cells), suggesting that everolimus enhanced sorafenib-induced apoptosis of MPM cells.

### Sorafenib and everolimus induce ROS production and cJun/p38-mediated apoptosis in MPM cell lines

**Figure 7:**
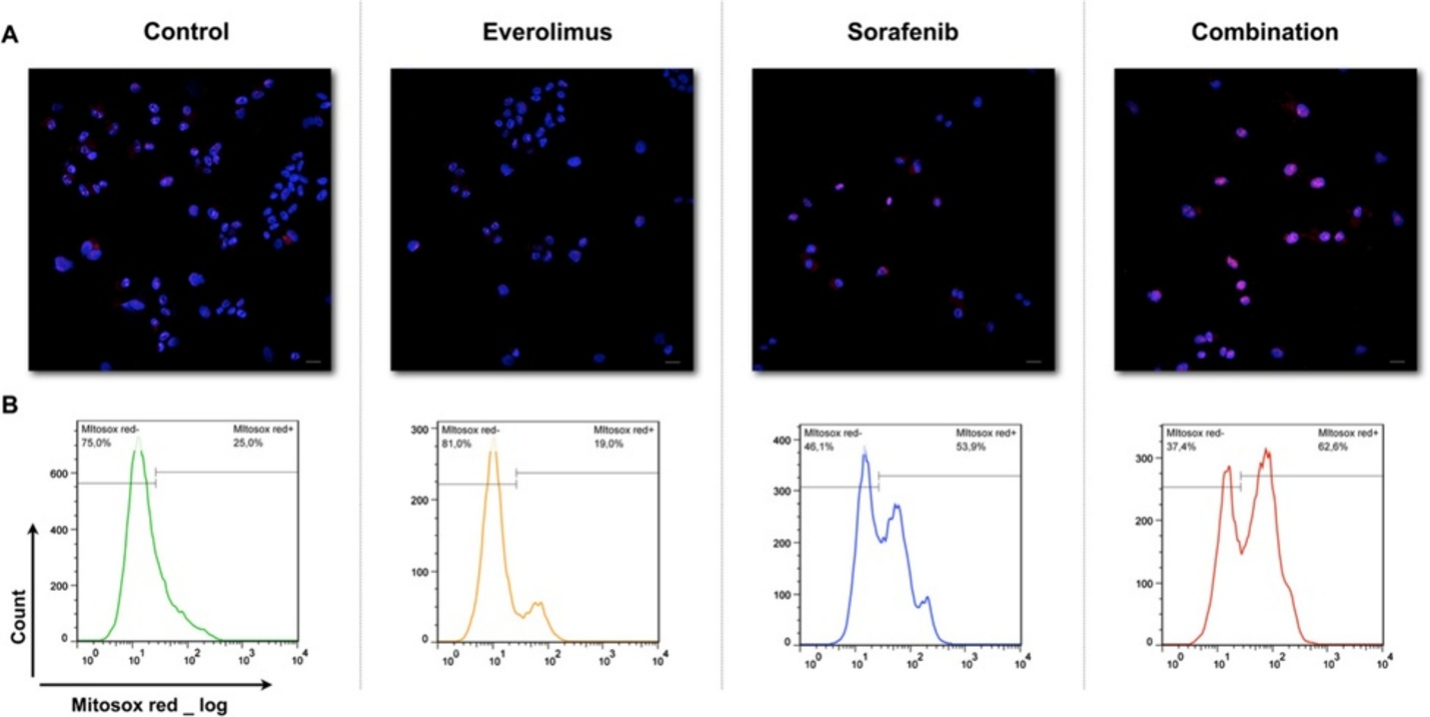
ROS production in MES-MM98 cells left untreated (control) or treated with everolimus 10 nM, sorafenib 5 μM and their combination for 24 h and then stained with the ROS-specific MitoSOX™ probe. **(A)** Confocal microscopy photomicrographs. Nuclei are counterstained with Hoechst 33342 bar = 10 μm. **(B)** Histograms of flow cytometry analysis.

To dissect the molecular pathways driving the apoptotic response in sorafenib- and everolimus-treated MES-MM98 cells, we first evaluated the phosphorylation of AMP-activated protein kinase (AMPK) on Thr172, an event that has been related to cellular stress and to the generation of mitochondrial ROS [20]. We observed that AMPK phosphorylation is specifically increased as a consequence of sorafenib treatment, both as a single agent and in the combination schedule (Figure 6C). We quantified ROS production by the use of a specific probe (MitoSOX™), demonstrating by confocal analysis that the percentage of positive cells dramatically increased in sorafenib (31.33 ± 4.3%) and combination-treated cells (55.98 ± 6.1%) in comparison with everolimus- treated cells (19.05 ± 2.7%) and untreated controls (16.22% ± 2.1%). These results were further confirmed by flow cytometry, allowing a better quantification of the percentage of cells with more brilliant signals produced by ROS and probe reactions (Figure 7). We also observed that, following a 15-minute pre-treatment and a further incubation in the presence of the ROS scavenger BHA, the percentage of apoptotic cells was significantly reduced in comparison to combination-treated cells (Figure 6B).

ROS have been traditionally viewed as generic cell-damaging agents; however, they also function as specific activators of key cellular processes, among which the MAPK signal transduction cascades [21]. Indeed, the stress-activated c-Jun and p38 MAPK have been implicated in the apoptotic response to different signals, including toxic chemical insults, environmental stress, and oxidative stress. We therefore evaluated the activation of these pathways by western blot analysis, observing that sorafenib alone and in combination with everolimus induced c-Jun and p38 activation (Figure 6C). This activation was correlated with the increase of apoptosis and ROS production mediated by these drugs (Figures 7B and 6). The apoptotic status was confirmed by the increase of PARP cleavage, the ultimate hallmark of apoptosis (Figure 6C). Inhibition of p38 pathway with the selective blocker SB202190 was paralleled by a reduction of apoptotic cells upon sorafenib and everolimus treatment confirming the role of p38 MAPK in sorafenib-everolimus induced apoptosis (Figure 6D).

### *In vivo* antitumor effects of sorafenib and everolimus on human MPM xenograft models

**Figure 8:**
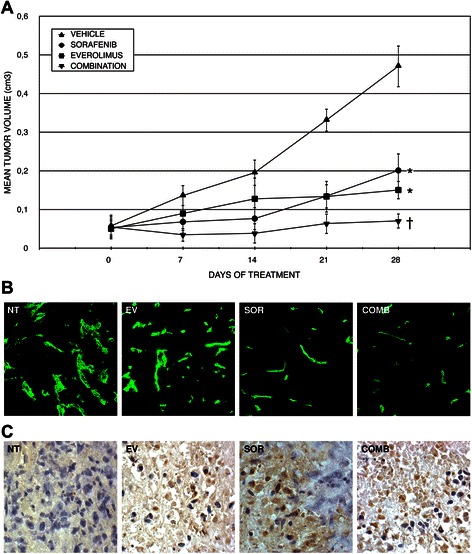
*In vivo* antitumor effect in human MPM xenograft models. **(A)** After tumor establishment, 6 mice per group were randomized to daily receive sorafenib 5 mg/kg/day, everolimus 1 mg/kg/day, their combination or vehicle alone. Tumor volume was monitored as described in the “material and methods” section and is reported in graph as mean ± St. Dev (Y error bar). *, p < 0.05 *vs* vehicle alone; †, p < 0.05 *vs* either single agent and vehicle alone. **(B)** Vascular specific CD31 staining and **(C)** TUNEL assay on vehicle (NT), everolimus 1 mg/kg/day (EV), sorafenib 5 mg/kg/day (SOR), and combination (CB) treated xenografts.

Based on the results obtained by *in vitro* experiments, we moved to assess a potential synergism of sorafenib and everolimus on preclinical models of human MPM obtained by subcutaneous injection of MPM cell lines in immunocompromised mice. We orally administered sorafenib (5 mg/kg/die) and everolimus (1 mg/kg/die) for 4 weeks, observing that both drugs induced a significant inhibition of tumour growth compared to the controls. The combination treatment reduced tumor growth at a higher extent, in comparison with both single drug alone or vehicle-treated controls (p < 0.05, Figure 8A). This effect was associated to increased numbers of apoptotic cells, as detected by TUNEL assay. The induction of apoptosis was slight after treatment with single-agent everolimus, significant after treatment with single-agent sorafenib, and reached the maximum following a combined therapy (Figure 8C). We finally evaluated the organization of vessels and capillaries by staining for the CD31 endothelial marker. This analysis revealed that sorafenib induced a sharp reduction of microvessels; conversely, everolimus was responsible of a weak impairment of the tumoral vessel network. The combination treatment resulted in an almost complete depletion of blood vessels into the tumoral area (Figure 8B). These data demonstrated that a combination of everolimus and sorafenib induced tumor cell apoptosis and reduced tumor vessels, thus proving to be a valuable therapeutic approach in preclinical models of human MPM.

## Discussion

MPM is an aggressive tumor with an ominous prognosis, due to the unavailability of effective therapeutic regimens. Albeit being considered a rare form of cancer, the incidence of MPM is on the rise, due to worldwide exposure to asbestos over the past decades. A better understanding of the molecular mechanisms involved in MPM carcinogenesis is urgently needed to design successful therapies that could offer MPM patients a real clinical benefit. The activation of mTOR pathway is a prognostic factor for MPM, in particular P-mTOR expression during induction chemotherapy was associated with shorter overall survival [8]. Moreover, mTOR inhibition has shown evidences of efficacy in MPM preclinical models [22,23]. In the present work, we confirmed that mTOR pathway is activated in MPM. With respect to the histologic subtype, in contrast with a previous report showing a prevalent mTOR expression in the epithelioid forms [8], we observed that the presence of an activated mTOR protein is unrelated to MPM histotypes. We further report that the phosphorylation of mTOR is not a consequence of the occurrence of somatic mutations in upstream mediators, e.g., PIK3CA, KRAS, BRAF and EGFR. These results suggest that, in MPM, the activation of mTOR signalling is triggered mainly by environmental and/or metabolic factors, among which a direct exposure to biopersistent fibers, such as asbestos [24]. We here demonstrated, for the first time to our knowledge, that pleural mesothelioma expresses activated ERM, and that the expression of its principal component ezrin is crucial for the motility and local aggressiveness of MPM cells. ERM proteins are cytoplasmic linkers between transmembrane proteins and the actin cytoskeleton with an active role in signal trasduction [11,12]. In particular, ezrin has been reported as responsible for cell survival signals transduced through the PI3K/AKT/mTOR pathway [25]. In a previous study we obtained ERM dephosphorylation by means of treatment with multikinase inhibitor sorafenib in preclinical models of osteosarcoma [17]. Based on these assumptions, we investigated whether a combined inhibition of mTOR pathway and sorafenib treatment could provide an effective strategy for MPM management. Despite a generally low efficacy of everolimus as a single agent, we reported a strong synergism of the two drugs in inhibiting cell proliferation *in vitro*. The combined treatment was capable to strongly induce apoptosis, by the induction of a ROS burst as we previously observed in sarcoma cell lines [26]. Consistently, we observed that sorafenib activates the energy sensor AMPK [27], further inducing mTOR pathway blockage. In the present work with mesothelioma cells, we further characterized apoptotic signalling induced by sorafenib and everolimus treatment. We demonstrated that sorafenib as single agent and even more in combination with everolimus induced mitochondrial ROS production, being this event related to apoptosis induction. Accordingly, treatment with a ROS scavenger protected MPM cells from apoptosis. Moreover, we observed p38 MAPK and c-Jun activation upon sorafenib and everolimus treatment inferring their specific role as downstream mediators of sorafenib and everolimus-induced apoptosis. In fact, we demonstrated that the inhibition with specific p38 chemical inhibitor SB202190 protected MPM cells from sorafenib- everolimus induced apoptosis. Again, according to previous results obtained in sarcoma models [17], we demonstrated that sorafenib was capable to inhibit also in MPM cells the phosphorylation of ERM, both as a single agent and in combination suggesting ERM as potential novel direct or indirect therapeutic targets of this drug. On the contrary, everolimus induced a weak activation of ERM when administered as a single agent. We hypothesized that this effect might be triggered by the activation of AKT [19,28] that, in turn, phosphorylates the tumor suppressor protein merlin [29]. In fact, this phosphorylation induced the dissociation of merlin from the complex with ezrin, moesin and CD44 allowing its switch toward an activated growth-promoting form [30]. Being merlin an interesting biomarker in MPM [31], reciprocal ezrin and merlin coordination in MPM onset and their influence on response to drugs deserve further investigations. The mTOR pathway is involved in ezrin- induced malignant phenotype [15], moreover the mTOR inhibition has shown antitumor effect in mesothelioma preclinical models [5,6,22,23]. To further investigate the effect of combining sorafenib and everolimus treatment in *in vivo* models of MPM, we set up MSTO-H211 xenografts into NOD/SCID mice. We observed that low doses of sorafenib and everolimus (physiologically achievable in human plasma and capable of inducing significant pharmacodynamic effect), as single agents and in combination, significantly reduced tumor growth without impairing the general health conditions of the treated mice. At the end of the experiments, no sign of adverse events was seen after necroscopy. Moreover, it will be important to investigate this treatment against tumors grown orthotopically to obtain insights on the effect on specific MPM microenvironment.

## Conclusions

In conclusion, everolimus and sorafenib exert antitumor activity in preclinical models of human MPM. These results provide a rationale for further clinical investigations, encouraging the planning of phase II trials with a combined sorafenib-everolimus schedule in MPM patients.

## Additional files

Additional file 1: Table S1. Clinico-pathological details for each analyzed case. Histology. E denotes epithelioid forms, S denotes sarcomatous forms, B denotes biphasic subtypes.
Additiona file 2: Figure S1. Correlation between outcome and mTOR and ezrin activation. The level of protein activation is expressed according the IHC staining. Data have been obtained through GraphPad analysis.
Additional file 3: Figure S2. Clonogenic properties of treated cells. Photograph of a representative colony growth of eight cell lines treated after 10 day-incubation in complete medium alone (NT) or with everolimus (EV 10 nM), sorafenib (SOR 1.25 μM), or their combination (SOR 1.25 μM + EV 10 nM) I cannot be able to see the addictional files in the proofs downloaded.

## Abbreviations

MPM: Malignant Pleural Mesothelioma
PIK3CA: Phosphatidylinositol-4,5-bisphosphate 3-kinase, catalytic subunit alpha
mTOR: Mammalian target of rapamycin
MAPK: Mitogen-activated protein kinases
ERM: Ezrin/radixin/moesin
EGFR: Epidermal growth factor receptor
AMPK: Adenosine monophosphate -activated protein kinase
NOD/SCID: Mice non obese diabetic /Severe combined immunodeficient mice
Thr: Threonine
SOR: Sorafenib
EV: Everolimus
DMSO: Dimethyl sulfoxide
s.c.: Subcutaneously
OS: Overall survival
P-mTOR: Phosphorylated mTOR
P-MAPK: Phosphorylated MAPK
P-ERM: Phosphorylated ERM
RSK: Ribosomal s6 kinase
p70S6K: p70S6 kinase
PI: Propidium iodide
DNA: Deoxyribonucleic acid
ROS: Reactive oxygen species
BHA: Butyl-4-hydroxyanisole
PARP: Poly (ADP-ribose) polymerase
ADP: Adenosine diphosphate
TUNEL: Terminal deoxynucleotidyl transferase dUTP nick end labeling
UTP: Uridine triphosphates
PI3K: Phosphatidylinositol-4,5-bisphosphate 3-kinase
NT: Untreated
CB: Combination
IC50: Concentration inhibiting 50% cell viability
CI: Combination index
Est.: St.dev estimanted standard deviation
