# Supplementary material for: The combination of sorafenib and everolimus shows antitumor activity in preclinical models of malignant pleural mesothelioma
Source: BMC Cancer. 2015 May 8;15:374. doi: 10.1186/s12885-015-1363-1 (PMC4429519; doi:10.1186/s12885-015-1363-1)
Supplement: Additional file 1: Table S1. — Clinico-pathological details for each analyzed case. Histology. E denotes epithelioid forms, S denotes sarcomatous forms, B denotes biphasic subtypes. [file 12885_2015_1363_MOESM1_ESM.docx]

**Supplementary Section**

**MPM samples selection**

We selected and analyzed 30 specimens from a cohort of patients aged ≥ 18 years who referred to the Pneumology Department at Fondazione IRCCS Policlinico San Matteo (Pavia, Italy) and who were subsequently diagnosed with MPM after medical thoracoscopy. Of them, 10 cases were epitheliod, 10 mesenchymal and 10 biphasic histological subtypes. Bioptic samples were evaluated at the Pathology Unit of the same hospital, and for each case formalin-fixed paraffin-embedded (FFPE) samples were obtained for immunohistochemistry (IHC) and molecular analysis. Complete clinical data of each patient studied are listed in Supplementary Table 1.

All the selected cases presented, after exhaustive staging, an advanced disease; they were consequently referred to conventional chemotherapy according to international guidelines^[[1]](#endnote-1),^^[[2]](#endnote-2),^^[[3]](#endnote-3)^ .

The vast majority of the patients were treated with cisplatin 75

mg

m^−2^ i.v. plus pemetrexed 500 mg

m^−2^ i.v. once every 3 weeks, for a maximum of 6 cycles, 8 cases were treated with the platinum-gemcitabine or with the addition of vinorelbine. Clinical response was assessed every 6 weeks with computed tomography (CT) scan according to the Response Evaluation Criteria in Solid Tumors (RECIST)^^[[4]](#endnote-4)^^. Three patients underwent palliative radiotherapy and one patient underwent surgery after chemotherapy.


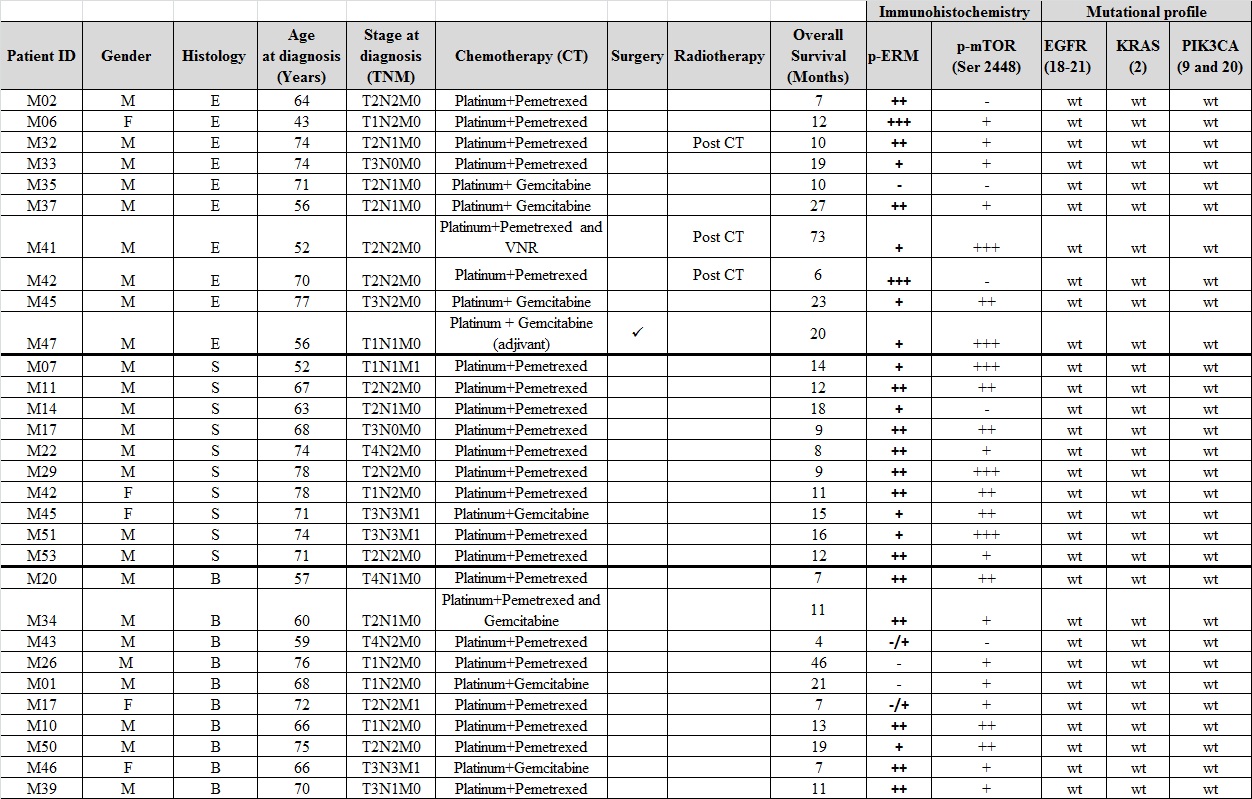


**Supplementary Table 1. Clinico-pathological details for each analyzed case.** Histology. E denotes epithelioid forms, S denotes sarcomatous forms, B denotes biphasic subtypes.

**Immunohistochemistry**

# The expression analysis of phospho-m-TOR and phospho-ERM and PCNA proteins was performed on four-micron FFPE MPM specimens from patients and xenografts mounted onto Superfrost Plus Microscope slides (Thermo Scientific, Braunschweig, Germany), dried in an oven at 60°C for 2 hours, deparaffinized in xylene, and rehydrated in graded alcohols and distilled water. Sections were heated in 10 mM citrate buffer pH 6.0 in a water bath at 96°C for 70 and 35 minutes, respectively, cooled, and stored in TBS at pH 7.6. Slides were incubated at room temperature with primary antibodies purchased by Cell Signaling Technologies (Danvers, MA, USA): Phospho-mTOR (Ser2448) (49F9) Rabbit mAb (IHC Specific) #2976; Phospho-Ezrin (Thr567)/Radixin (Thr564)/Moesin (Thr558) (41A3) Rabbit mAb#3149, and PCNA (D3H8P) XP® rabbit mAb #13110 diluted according to manufacturer’s instructions. Endogenous peroxidase activity was blocked with 0.3% hydrogen peroxide for 20 minutes at room temperature. The reactions were revealed with the ImmPRESS (Vector Laboratories, Burlingame, CA, USA) detection system, using diaminobenzidine tetrahydrochloride as chromogen substrate (Dako Carpinteria, CA, USA). Samples were counterstained with Harris’hematoxylin (VWR, Poole, England) for 1 minute, dehydrated in a series of graded ethanols, cleared in xylene and mounted. Each reaction set included positive controls as suggested by the manufacturer and a negative control slide incubated exclusively with the dilution buffer. All the immunostained slides were examined at light microscopy by two independent observers S.I and P:M who recorded the cell types expressing each antigen and who semiquantitatively scored the intensity of protein expression. Cell staining intensity was graded as follows: no staining (0), weak (+), moderate (++), and intense (+++). In case of disagreement, slides were re-evaluated collectively to obtain a final agreement on the score (Suppl. Table 1).

**Mutational analysis**

From each FFPE sample, tumor DNA was extracted by using a commercial kit (Nucleospin Tissue, Macherey-Nagel Neumann-Neander-Straße, Düren, Germany), following the manufacturer’s recommendations. We checked the mutational profile of ‘hot spot’ regions of three oncogenes frequently mutated in solid cancers: KRAS (exon 2), EGFR (exons18-19-20-21) and PIK3CA (exons 9-20). Each exon was individually PCR-amplified, and the PCR products were directly sequenced bidirectionally by dye-terminator sequencing after PCR purifications with Ampure Magnetic Beads (Agencourt, Beverly, Mass). Sequencing products were purified using Cleanseq Magnetic Beads (Agencourt, Beverly, Mass) and separated by capillary electrophoresis on a CEQ 8800 DNA Analyzer (Beckman-Coulter Cassina de Pecchi (MI), Italy). Primers for PCR and sequence are summarized in Supplementary Table 2. Sequence data were analyzed using a Bioedit^[[5]](#endnote-5)^ and manually reviewed.

**Supplementary Table 2.** Exons and primers used for mutational analysis for each gene in study.

**MPM cell culture**

Cells were cultured as monolayers into tissue culture treated Petri dishes using RPMI 1640 or Ham’s F12 (Invitrogen, Paisley, UK) (for HMM-1 and HMM-2) in the presence of 10% heat inactivated fetal bovine serum (FBS), 1% L-glutamine and penicillin/streptomycin (10,000 U/ml and 10,000 g/ml, respectively), with medium changes every 2 to 3 days and maintained in humidified atmosphere containing 5% CO_2_ at 37°C.

***In vitro* Viability Assay**

Each MPM cell line was examined with 1:2 scalar doses of SOR (from 10μM to 0.625μM) and EV (from 2μM to 125nM). 3000 cells/well were plated in 96-well plates with 100μl of their specific culture medium. After 72 h, cell proliferation was evaluated with the Cell Titer-Glo^®^ luminescent cell viability kit (Promega Corporation, Madison, WI, USA). A volume of 100µl of the reaction reagent was added to each well, and after 10 min the luminescence signal was detected using GLOMAX 96 Microplate Luminometer (Promega, Madison, WI). Cell survival fractions were also evaluated by the MTT (3-[4,5-dimethylthiazol-2-yl]-2,5-diphenyltetrazoliumbromide) dye reduction assay (Sigma-Aldrich). After drug treatment, cell survival fractions were incubated with MTT for 3 hours and analyzed using GLOMAX 96 Microplate Luminometer (Promega, Madison, WI). All the experiments were repeated at least three times. The IC50 value (concentration of each drug inhibiting 50% cell proliferation) and synergism (sorafenib+everolimus) were assessed through normalized isobologram and combination index (CI) by CalcuSyn software (BIOSOFT, Great Shelford – Cambridge, UK).

**Colony forming assay**

The clonogenic assay enables an assessment of the differences in reproductive viability (capacity of cells to produce progeny; i.e. a single cell to form a colony of 50 or more cells) between control, untreated cells and cells that have undergone a treatment. 250-300 cells/well were plated in 12-well plates. After the formation of colonies (derived from single cells), cells were treated with scalar doses of sorafenib (from 5 to 1.25μM) and with everolimus (10nM), either as single agent or in combination. Medium was replaced every 72 h. After 10-15 days, the colonies were stained with crystal violet. The effects of each treatment were established with the BD pathway HT Bioimager System calculating the colony number and the area occupied by the colonies on the well.

**Western blot analysis**

MPM cells (80% confluence) were treated for 24h with EV (100 nM) or with SOR (5 μM), alone or in combination, or left untreated. Five to ten million cells were washed with 1× PBS and lysed in boiling buffer (which contained SDS 10%, TRIS HCl 0.5M pH6.8) at 100°C. The samples were boiled for 5 min, sonicated for 20 sec, and then centrifuged at 14,000 rpm for 30 minutes. The protein concentration of cell lysates was measured using the BCA Protein Assay (Thermo Scientific, Rockford, IL, USA) and 20-50 μg of proteins were resolved by 6-15% SDS-polyacrylamide gel electrophoresis and electrotransferred to nitrocellulose membranes (Amersham Pharmacia Biotech, Piscataway, NJ, USA) at 110 mA for 70 minutes at 4°C. Nonspecific sites were blocked by incubating for 1 hour with 5% non-fat dry milk (Bio-Rad Laboratories, Hercules, CA, USA) in Tris-buffered saline-Tween (TBST) (20 mM Tris-HCl pH 7.5, 500 mM NaCl, 0.1% Tween-20). Membranes were incubated overnight with the primary antibodies: phospho-Ezrin (Thr567)/Radixin (Thr564)/Moesin (Thr558) (41A3) Rabbit mAb #3149; Ezrin Antibody #3145; Phospho-p90RSK (Ser380) Antibody #9341; RSK1/RSK2/RSK3 (32D7) Rabbit mAb #9355; phospho-p44/42 MAPK (Erk1/2) (Thr202/Tyr204) (D13.14.4E) XP® Rabbit mAb #4370; Phospho-Akt (Ser473) (D9E) XP® Rabbit mAb #4060; Akt Antibody #9272; Phospho-4E-BP1 (Ser65) Antibody #9451; 4E-BP1 (53H11) Rabbit mAb #9644; Phospho-mTOR (Ser2448) (D9C2) XP® Rabbit mAb #5536; mTOR (7C10) Rabbit mAb #2983; p38 MAPK Antibody #9212; Phospho-p38 MAPK (Thr180/Tyr182) (D3F9) XP® Rabbit mAb #4511; Phospho AMPKα (Thr172) Antibody #2531; AMPKα (23A3) Rabbit mAb #2603; Phospho-c-Jun (Ser63) II Antibody #9261; c-Jun (60A8) Rabbit mAb #9165; cleaved PARP (Asp214) Antibody (Human Specific) #9541 were purchased by Cell Signaling Technologies (Danvers, MA, USA); Vinculin ( V9131) was purchased from Sigma Aldrich Italia (Milano , Italy); [ERK 1/2 (H-72)](http://www.scbt.com/datasheet-292838-erk-1-2-h-72-antibody.html) was from Santa Cruz Biotechnology (Dallas, Texas, USA). After washing with TBST, blots were incubated with the appropriate peroxidase-conjugated secondary antibody for 1 h at room temperature and the specific signals were developed by ECL (LiteAblot TURBO Euroclone, Pero, IT) according to manufacturer’s instructions. The immunoreactive bands were quantified with the Quantity One Version 4.6.0 analysis software (Bio-Rad), using the Vinculin protein signals as a normalizer.

**Annexin V/PI determination of apoptosis and ROS analysis**

# MPM cells were plated in 100 mm-dishes with appropriate culture medium. After 24h medium was replaced with fresh completed medium with sorafenib (2.5µM) and everolimus (10nM), alone and in combination. After 72 hours of incubation at 37°C, both adherent and non-adherent cells were collected, washed once in cold PBS and twice in binding buffer (150 mM NaCl_2_, 10 mM CaCl_2_, 10 mM Hepes). Cells were centrifuged at 3,000 rpm for 5 min, resuspended in binding buffer and incubated with APC-labelled Annexin V (eBioscence, Wien, AT) and PI (0.5 µg/ml) for 15 min at RT in the dark. The samples were analyzed by Cyan ADP Flow cytometer using Summit v4.3 software. To test ROS production, MPM cells were cultured to 70-80% confluence, followed by drug treatment or by incubation with H_2_O_2_ (as a positive control) for 1 h. Harvested cells were incubated for 30 min with 10 µM carboxy-H2-DCFDA or 10 µM MitoSOX™ Red (Molecular Probes, Carlsbad, CA), washed twice in 1% BSA in PBS, and fluorescence was analyzed by flow cytometry. In selected experiments, apoptosis and ROS production were tested concomitantly by combining carboxy-H2-DCFDA with annexinV and PI staining in a unique 30-minute incubation. Cells were also cultured onto Chamber slides (Nunc® Lab-Tek® II Chamber Slide™ system) and, following drug treatment, they were double-stained with MitoSOX Red (Molecular Probes, Life technologies) and Hoechst 33342 (Sigma Aldrich) and analysed with a laser confocal Leica TCS SP5 microscope (Leica Microsystems Srl, Milano, Italy)

**Cell cycle analysis**

Cells were cultured with appropriate culture medium containing 10% FBS. Cells were serum-starved for 24 h prior to drugs exposure, followed by treatment with sorafenib (2.5μM) and everolimus (10nM), alone and in combination, in fresh complete medium. Cells were dissociated with trypsin, washed in cold PBS and fixed with 70% cold ethanol at -20°C. After 24 h cells were washed twice in PBS and incubated in PI staining solution (PI 50μg/ml and RNase 100μg/ml dissolved in PBS) for 3 h on ice in the dark. Cyan ADP Flow cytometer was used to analyze the cell cycle distribution. Approximately 30,000 cells were examined for each sample. The percentage of cells in the G0/G1, S and G2/M phase of the cell cycle were determined using Summit v4.3 software.

**Silencing of EZRIN**

MPM cells were seeded, 120,000 per well in 6-well plates, and after 24 h medium was replaced with Opti-MEM® serum-free medium (Gibco, USA). Two mixes were prepared: the first containing 5nmol ezrin (EZR) specific siRNA sense 5’GGUUUCCUUGGAGUGAAtt3’ and antisense 5’UUUCACUCCAAGGAAAGCCaa3’) or control siRNA (AllStars Negative Control siRNA- Qiagen, Germany) and the second containing oligofectamine (Invitrogen, Carlsbad, USA) and Opti-MEM®. These solutions were maintained for 5 minutes at room temperature, then combined and incubated for 20 min at RT. Transfection of MPM cells with siRNAs was performed using 10 μl of siRNAs against ezrin (20 μmoli/L) formulated with 4 μl of oligofectamine, applied at the final volume of 1 ml. Cells were incubated for 6 hours and then medium was replaced following the supplier's instructions. To check the silencing efficiency, the expression levels of the ezrin protein were analyzed by western blot 24, 48 and 72 h after transfection.

**Scratch assay**

To evaluate cell migration, confluent monolayers of MPM cells (either parental or siRNA transfected) were scratched with a pipette tip across the monolayer. The cells were washed with PBS to remove loose cells and were cultured in RPMI-1640 (2% FBS) without or with tested drugs (parental MPM cells). Images were obtained using the BD pathway HT Bioimager System immediately after the wounding and then again at 24 h post-wounding. Cell migration was measured by ImageJ software, calculating the differences of wound height after different treatments or between control siRNA trasfected cells and EZRIN-specific siRNA transfected cells. The observed cell migration inhibition was confirmed by using the CIM-Plate 16 with the RTCA DP Instrument (Roche). The CIM-Plate was assembled following the manufacturer’s instructions, using media containing 10% bovine serum as a chemotaxis inducer. Cells were serum-starved for 2 hours prior to detachment for migration assay. The cells were collected and washed after their trypsinization, resuspended in serum free medium and treated with sorafenib (5μM) and everolimus (100nM) alone and in combination. Measurements were stopped after 24 hours and analyzed with the RTCA software evaluating the Cell Index curve.

**Mice Xenograft models**

Non-obese diabetic/severe combined immunodeficient (Nod/SCID) female mice (Charles River, Italia) were breed, maintained in cage microinsulators, and handled under sterile conditions at the animal facilities of Comparative Oncology Center (COC) at the Institute for Cancer Research and Treatment and treated in accordance with and approved by the Ethical Commission of the Institute for Cancer Research and Treatment (Candiolo, Torino, Italy), and of the Italian Ministry of Health.

In three different experiments, 24 mice (4-6 weeks old) were injected subcutaneously (s.c.) into the right flank with 10^6^ MSTO-211H cells in 50% growth factor-reduced BD Matrigel basement membrane matrix (BD Biosciences, San Jose, CA). When xenografts were established at about 100 mm^3^ after 5 weeks, animals (divided into four groups of five mice) were treated daily by oral gavage with sorafenib (5 mg/kg/die), everolimus (1 mg/kg/die), and their combination (sorafenib 5mg/kg/die + everolimus 1 mg/kg/die) or vehicle alone for 4 weeks and then sacrificed.

Tumor diameters were measured at the beginning of the treatment and then every 7 days using callipers; volumes (V) were calculated using the following formula: V = A*B^2^/2 (A = largest diameter; B = smallest diameter). Mean volumes of treated and untreated xenografts were compared using an unpaired t test (Student’s t test), considering as statistically significant a p value <0.05. Tumors were processed for further protein and nucleic acid extractions, and for histological and immunohistochemical evaluations after immediate stocking in liquid nitrogen (two fourths), in 10% formalin (one forth), in OCT medium (one fourth), respectively.

Apoptotic cells were detected by the terminal deoxynucleotidyl transferase (TdT)-mediated by dUTP-biotin nick end labeling (TUNEL) method, using the commercial kit ApopTag® plus peroxidase in situ apoptosis detection kit (Chemicon, CA, USA; code S7101), following the manufacturer’s instructions. Briefly, 3-μm sections of paraffin-embedded tissue were subjected to deparaffination, hydration with ethanol, incubation with proteinase K, blockage of endogenous peroxidase activity with a solution of 3% hydrogen peroxide in methanol, incubation with the equilibration buffer, incubation with the working strength TdT enzyme, incubation with the stop/wash buffer, and incubation with the anti-digoxigenin conjugated. Color development was performed with diaminobenzidine. Slides were counterstained with hematoxylin. Omission of the TdT enzyme in the TUNEL reaction was used as a negative control. CD31 staining of microvessels was done following conventional immunofluorescence protocol with primary antibody purchased by Sigma-Aldrich (clone WM-59 )mouse monoclonal antibody anti- CD31 (PECAM-1) catalog number P8590.

1. NCCN Clinical Practice Guidelines in Oncology , website at [www.nccn.org](http://www.nccn.org) [↑](#endnote-ref-1)
2. Scherpereel A, Astoul P, Baas P, Berghmans T, Clayson H, de Vuyst P, Dienemann H, Galateau-Salle F, Hennequin C, Hillerdal G, Le Péchoux C, Mutti L, Pairon JC, Stahel R, van Houtte P, van Meerbeeck J, Waller D, Weder W. Guidelines of the European Respiratory Society and the European Society of Thoracic Surgeons for the management of malignant pleural mesothelioma. [*Eur Respir J*. 201;35(3):479-95.](http://erj.ersjournals.com/cgi/content/full/35/3/479) [↑](#endnote-ref-2)
3. [Stahel](http://annonc.oxfordjournals.org/search?author1=R.+A.+Stahel&sortspec=date&submit=Submit) RA, [Weder](http://annonc.oxfordjournals.org/search?author1=W.+Weder&sortspec=date&submit=Submit) W, [Lievens](http://annonc.oxfordjournals.org/search?author1=Y.+Lievens&sortspec=date&submit=Submit) Y, [Felip](http://annonc.oxfordjournals.org/search?author1=E.+Felip&sortspec=date&submit=Submit) E. Malignant pleural mesothelioma: ESMO Clinical Practice Guidelines for diagnosis, treatment and follow-up. *Ann Oncol* 2010; 21 (suppl 5): v126-v128 [↑](#endnote-ref-3)
4. [Therasse P](http://www.ncbi.nlm.nih.gov/pubmed?term=%22Therasse%20P%22%5BAuthor%5D), [Arbuck SG](http://www.ncbi.nlm.nih.gov/pubmed?term=%22Arbuck%20SG%22%5BAuthor%5D), [Eisenhauer EA](http://www.ncbi.nlm.nih.gov/pubmed?term=%22Eisenhauer%20EA%22%5BAuthor%5D), [Wanders J](http://www.ncbi.nlm.nih.gov/pubmed?term=%22Wanders%20J%22%5BAuthor%5D), [Kaplan RS](http://www.ncbi.nlm.nih.gov/pubmed?term=%22Kaplan%20RS%22%5BAuthor%5D), [Rubinstein L](http://www.ncbi.nlm.nih.gov/pubmed?term=%22Rubinstein%20L%22%5BAuthor%5D), [Verweij J](http://www.ncbi.nlm.nih.gov/pubmed?term=%22Verweij%20J%22%5BAuthor%5D), [Van Glabbeke M](http://www.ncbi.nlm.nih.gov/pubmed?term=%22Van%20Glabbeke%20M%22%5BAuthor%5D), [van Oosterom AT](http://www.ncbi.nlm.nih.gov/pubmed?term=%22van%20Oosterom%20AT%22%5BAuthor%5D), [Christian MC](http://www.ncbi.nlm.nih.gov/pubmed?term=%22Christian%20MC%22%5BAuthor%5D), [Gwyther SG](http://www.ncbi.nlm.nih.gov/pubmed?term=%22Gwyther%20SG%22%5BAuthor%5D). New guidelines to evaluate the response to treatment in solid tumors. *[J Natl Cancer Inst.](http://www.ncbi.nlm.nih.gov/pubmed?term=J%20Natl%20Cancer%20Inst%202000%3B%2092%3A205-216.%20tumors)* 2000;92(3):205-16 [↑](#endnote-ref-4)
5. Hall, T.A. 1999. BioEdit: a user-friendly biological sequence alignment editor and analysis program for Windows 95/98/NT. Nucl. Acids. Symp. Ser. 41:95-98 [↑](#endnote-ref-5)
