# Supplementary material for: The combination of sorafenib and everolimus shows antitumor activity in preclinical models of malignant pleural mesothelioma
Source: BMC Cancer. 2015 May 8;15:374. doi: 10.1186/s12885-015-1363-1 (PMC4429519; doi:10.1186/s12885-015-1363-1)
Supplement: Additiona file 2: Figure S1. — Correlation between outcome and mTOR and ezrin activation. The level of protein activation is expressed according the IHC staining. Data have been obtained through GraphPad analysis. [file 12885_2015_1363_MOESM2_ESM.docx]

Supplementary Figure 1. Correlation between outcome and mTOR and ezrin activation. The level of protein activation is expressed according the IHC staining. Data have been obtained through GraphPad analysis.
