# Supplementary figures and images for: The combination of sorafenib and everolimus shows antitumor activity in preclinical models of malignant pleural mesothelioma
Source: BMC Cancer. 2015 May 8;15:374. doi: 10.1186/s12885-015-1363-1 (PMC4429519; doi:10.1186/s12885-015-1363-1)

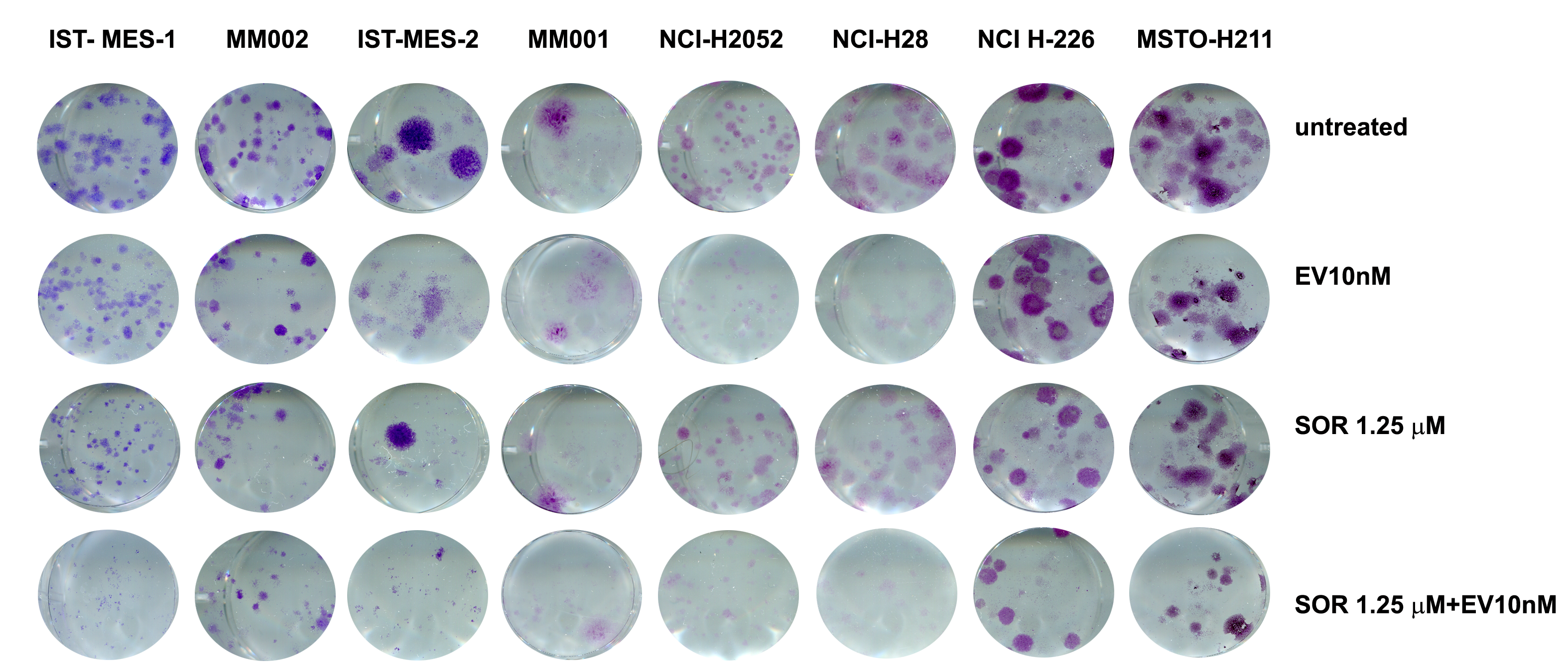

Supplement: Additional file 3: Figure S2. — Clonogenic properties of treated cells. Photograph of a representative colony growth of eight cell lines treated after 10 day-incubation in complete medium alone (NT) or with everolimus (EV 10 nM), sorafenib (SOR 1.25 μM), or their combination (SOR 1.25 μM + EV 10 nM) I cannot be able to see the addictional files in the proofs downloaded. [file 12885_2015_1363_MOESM3_ESM.tiff]
